# Antiproliferative Effects of Hop-derived Prenylflavonoids and Their Influence on the Efficacy of Oxaliplatine, 5-fluorouracil and Irinotecan in Human ColorectalC Cells

**DOI:** 10.3390/nu11040879

**Published:** 2019-04-19

**Authors:** Martin Ambrož, Kateřina Lněničková, Petra Matoušková, Lenka Skálová, Iva Boušová

**Affiliations:** 1 Faculty of Pharmacy in Hradec Králové, Charles University, Heyrovského 1203, 500 05 Hradec Králové, Czech Republic; ambrozm@faf.cuni.cz (M.A.); matousp7@faf.cuni.cz (P.M.); skaloval@faf.cuni.cz (L.S.); 2 Faculty of Medicine and Dentistry, Palacký University, Hněvotínská 3, 775 15 Olomouc, Czech Republic; katerina.lnenickova@upol.cz

**Keywords:** colorectal carcinoma cells, irinotecan, oxaliplatin, 5-fluorouracil, prenylflavonoids, naringenin, isoxanthohumol, caspase activity

## Abstract

Beer, the most popular beverage containing hops, is also frequently consumed by cancer patients. Moreover, non-alcoholic beer, owing to its nutritional value and high content of biological active compounds, is sometimes recommended to patients by oncologists. However, the potential benefits and negatives have to date not been sufficiently evaluated. The present study was designed to examine the effects of four main hop-derived prenylflavonoids on the viability, reactive oxygen species (ROS) formation, activity of caspases, and efficiency of the chemotherapeutics 5-fluorouracil (5-FU), oxaliplatin (OxPt) and irinotecan (IRI) in colorectal cancer cell lines SW480, SW620 and CaCo-2. All the prenylflavonoids exerted substantial antiproliferative effects in all cell lines, with xanthohumol being the most effective (IC_50_ ranging from 3.6 to 7.3 µM). Isoxanthohumol increased ROS formation and the activity of caspases-3/7, but 6-prenylnaringenin and 8-prenylnaringenin exerted antioxidant properties. As 6-prenylnaringenin acted synergistically with IRI, its potential in combination therapy deserves further study. However, other prenylflavonoids acted antagonistically with all chemotherapeutics at least in one cell line. Therefore, consumption of beer during chemotherapy with 5-FU, OxPt and IRI should be avoided, as the prenylflavonoids in beer could decrease the efficacy of the treatment.

## 1. Introduction

Colorectal carcinoma (CRC) is the third most common cancer in men and the second in women worldwide. It also represents the fourth leading cause of cancer deaths in the world [1]. Depending on its type and stage, there are several ways to treat CRC, including local and systemic therapies. Local therapies of CRC comprise surgical resection, radiation therapy, ablation, and embolization; whereas chemotherapy, immunotherapy and targeted therapy rank among the systemic approaches [2]. While curative surgery remains the mainstay of treatment for early stage CRC, the therapy of patients with lymph node positive or the metastatic form of CRC is primarily based on cytotoxic chemotherapy or radiochemotherapy. Fluoropyrimidines (e.g. 5-fluorouracil, capecitabine), irinotecan, oxaliplatin, and combinations of these drugs are used in CRC chemotherapy, with 5-fluorouracil being the cornerstone. In spite of advances in systemic therapy, treatment failure occurs in almost 90% of patients with metastatic cancer. Intrinsic or acquired resistance to chemotherapy is likely to be a cause of the treatment failure [3,4]. Nevertheless, undesirable interactions of chemotherapeutics with other drugs and/or food components might also decrease the efficacy of the therapy; thus, to identify the food components which can either enhance or impair the efficacy of traditional chemotherapeutic regimens is of great importance.

Flavonoids, a group of polyphenolic secondary plant metabolites, exert numerous biological and pharmacological activities including anti-cancer properties [5]. A positive correlation between a flavonoids-rich diet and the lower risk of CRC has been reported [6]. Hops (strobili of *Humulus lupulus* L., Cannabaceae) are a unique source of natural prenylated flavonoids such as xanthohumol, its isomer isoxanthohumol; 8-prenylnaringenin; and 6-prenylnaringenin. These prenylflavonoids exhibit e.g. antioxidant [7], anti-viral and antibacterial [8], antidiabetic [9], anti-inflammatory [10], and estrogenic activities [11]. Previous investigations have shown that xanthohumol as well as other prenylflavonoids exert antitumor activity in multiple tumors such as CRC, breast cancer, glioblastoma and pancreatic cancer by inducing cell apoptosis [12,13,14] and inhibiting angiogenesis [15]. Moreover, isoxanthohumol enhances the cytostatic effect of paclitaxel on the melanoma cells in vitro as well as in vivo [16]. 

Beer, a frequently consumed hop-containing beverage, is the most important dietary source of prenylflavonoids. The chemopreventive activity of beer and its constituents including prenylflavonoids has been studied and reported repeatedly. In view of these facts, the reasonable consumption of beer seems to be beneficial for all, particularly for cancer patients. Some oncologists even recommend non-alcoholic beer to patients undergoing chemotherapy [17,18], although the potential benefits and negatives have not been sufficiently evaluated yet.

**Figure 1:**
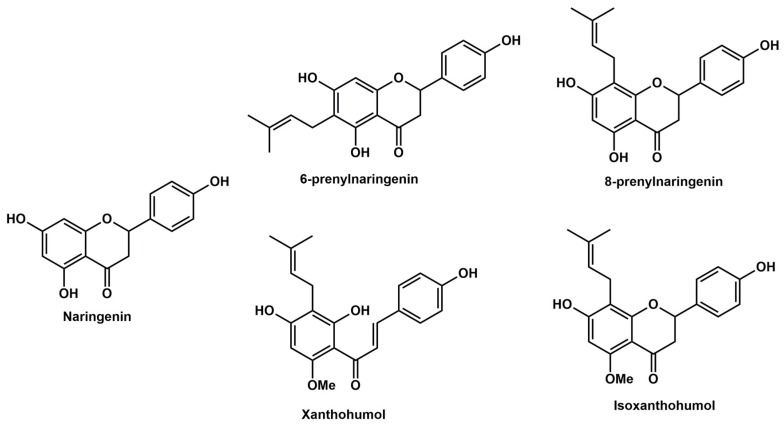
Chemical structures of the studied prenylflavonoids and naringenin.

In the present study, the effects of prenylflavonoids xanthohumol (XH), isoxanthohumol (IXH), 6-prenylnaringenin (6-PN), and 8-prenylnaringenin (8-PN) on cell proliferation were studied and compared in four colorectal cell lines. For this purpose, two isogenic human cell lines were used, one established from a colorectal adenocarcinoma (SW480) and the other from its lymph node metastasis (SW620) along with proliferating as well as differentiated CaCo-2 cells, both lines derived from human epithelial colorectal adenocarcinoma. Moreover, the ability of these prenylflavonoids to influence the efficacy of the classical chemotherapeutics 5-fluorouracil (5-FU), irinotecan (IRT) and oxaliplatin (OxPt) in SW480 and SW620 cell lines in vitro was studied. The effect of the prenylflavonoids was compared to that of naringenin (NAR), their structural analogue lacking the prenyl group. We hypothesize that prenylflavonoids could enhance the efficacy of classical chemotherapeutics in colorectal cancer cells probably via increasing reactive oxygen species (ROS) formation and/or induction of caspases. The structures of studied flavonoids are presented in Figure 1.

## 2. Materials and Methods 

### 2.1. Chemicals and Reagents

Dulbecco’s Modified Eagle Medium (DMEM) high glucose and Eagle’s Minimum Essential Medium (EMEM) were obtained from Biosera (Nuaille, France). Xanthohumol (XH), isoxanthohumol (IXH), 6-prenylnaringenin (6-PN) 8-prenylnaringenin (8-PN), naringenin (NAR), oxaliplatin (OxPt), 5-fluorouracil (5-FU), irinotecan (IRT), N-2-hydroxyethylpiperazine-N′-2-ethanesulfonic acid (HEPES) buffer, phosphate-buffered saline (PBS), 3-[(3-Cholamidopropyl)dimethylammonio]-1-propanesulfonate hydrate (CHAPS), DL-dithiothreitol (DTT), and neutral red were purchased from Sigma–Aldrich (Prague, Czech Republic). Fetal bovine serum (FBS) and gentamicin sulfate were supplied by Invitrogen (Carlsbad, CA, USA), and bovine serum albumin (BSA) by Fluka (Prague, Czech Republic). All other chemicals were of HPLC or analytical grade (Sigma–Aldrich, Czech Republic). Stock solutions of prenylflavonoids and naringenin were prepared in dimethyl sulfoxide (DMSO) and stored at 4 °C in the dark.

### 2.2. Cancer Cell Culture

Human colon cancer cell lines SW480 and SW620 as well as human epithelial colorectal adenocarcinoma CaCo-2 cell line were purchased from ATCC (supplier for Czech Rep.: LGC Standards, Poland). Cells were proliferated in three passages, frozen in aliquots and stored in liquid nitrogen. All the cell lines used in the laboratory are periodically tested for the mycoplasma contamination. For every set of experiments (lasting 3–9 weeks), new storage cells were revived. 

The SW480 and SW620 cells were maintained in DMEM medium supplemented with 10% heat-inactivated FBS, 1% HEPES and 0.5% gentamicin in a humidified atmosphere containing 5% CO_2_ at 37 °C and subcultured every 3 days. 

The CaCo-2 cell line was cultured in EMEM medium supplemented with 10% heat-inactivated FBS, 1% non-essential amino acids, 1% glutamine, and 0.5% penicillin/streptomycin. The cells were grown in 96-well plates for 3 (proliferating cells) and 21 (differentiated cells) days in a humidified atmosphere containing 5% CO_2_ at 37 °C and subcultured every 3 days. 

### 2.3. Cell Viability Test

The prenylflavonoids and NAR were pre-dissolved in DMSO, while 5-FU, IRT and OxPt were pre-dissolved in distilled water. The concentration of DMSO in medium did not exceed 0.2%. During screening for antiproliferative effects, SW480, SW620, and CaCo-2 were exposed to various concentrations of individual compounds in culture medium: XH, IXH, 6-PN and 8-PN (1–100 μmol/L), NAR (1–200 µmol/L), 5-FU and IRT (0.1–100 µmol/L), and OxPt (0.1–25 µmol/L). The cells cultured in medium with 0.1% DMSO or 0.2% DMSO (for 8-PN only) were used as untreated control samples. In the case of OxPt, the untreated control sample contained cells cultured in medium only. The cells treated with 10% DMSO served as a positive control. Neutral red uptake (NRU) assay was used to determine the number of viable cells after 72 h of exposition. The effect of prenylflavonoids and NAR on 5-FU, IRT or OxPt efficacy was evaluated using CalcuSyn (ver. 1.1) software.

In the NRU assay, the cells were cultured in 96-well plates (SW480 and SW620: 2500 cells/well; CaCo-2 cells: 200 cells/well) and exposed to the tested compounds for 72 h. Thereafter, the medium was removed and 100 μL of neutral red-containing medium was added into each well and the plates were incubated at 37 °C for an additional 3 h. Then the cells were washed with 100 μL of PBS and fixed in a solution of 0.5% formaldehyde/1% calcium chloride for 15 minutes. The neutral red dye was extracted from the viable cells with a solvent (50% ethanol/1% acetic acid) by shaking for 30 min at room temperature. The absorbance of solubilized dye was measured using the spectrophotometer Infinite M200 (Tecan, Männedorf, Switzerland) at 540 nm. Each sample was assayed in six parallels and three independent experiments were performed. The viabilities of treated cells were expressed as a percentage of untreated controls (100%). 

### 2.4. Caspases Activity Assay

The activities of initiator caspases-8 and -9 and effector caspases-3/7 were assayed using Caspase-Glo Assays (Promega, Madison, USA). The SW620 cells (seeded in a 96-well plate at a density of 30,000 cells/well) were cultivated in 12-well plates. After 4, 8 and 24 h incubation of cells in the medium containing the tested compounds pre-dissolved in DMSO at a concentration equal to their IC_50_ concentration, cells were lysed using a buffer containing 50 mM HEPES, 5 mM CHAPS and 5 mM DTT. The untreated control cells were incubated in medium containing only 0.1% DMSO. The lysates were collected into microtubes. Caspase-8, Caspase-9 and Caspases-3/7 reagents were prepared according to the manufacturer’s instructions. The lysates (25 μL in each well) were transferred into a white-walled 384-well-plate for the luminometer, at which point 25 μL of Caspase-8 or Caspase-9 or Caspases-3/7 reagents were added to each well. The plate was gently mixed for 30 seconds and then incubated for 30 minutes at room temperature. The luminescence was measured using the luminometer Infinite M200 (Tecan, Männedorf, Switzerland).

### 2.5. Measurement of Cellular ROS Formation

To assess ROS generation in SW620 cells, a measurement of 2′,7′-dichlorodihydrofluorescein-diacetate (H_2_DCF-DA) oxidation was used. Cells (seeded in a 96-well plate at a density of 2500 cells/well) were washed with PBS buffer and incubated with 100 μL of 5 μM H_2_DCF-DA in Hank’s balanced salt solution (HBSS). Then the cells were washed with PBS buffer, and 100 μL of the tested compounds at a concentration equal to the IC_50_ value (individual prenylflavonoids/NAR, 5-FU/IRT/OxPt alone, 5-FU/IRT/OxPt + prenylflavonoid/NAR combinations pre-dissolved in DMSO) in an HBSS buffer were added and incubated for 0.5, 4, 8, and 24 h. Finally, fluorescence intensity was measured at the excitation and emission wavelengths of 485 nm/525 nm for 50 min at 37 °C using the microplate reader Infinite M200 (Tecan, Männedorf, Switzerland). The cells incubated in HBSS with DMSO only (as a solvent) were used as untreated control samples, while those incubated in the presence of 3% H_2_O_2_ were used as a positive control.

### 2.6. Statistical Analysis

All calculations were done using Microsoft Excel and GraphPad Prism 7.03 (GraphPad Software, La Jolla, CA, USA). All values were expressed as mean ± S.D. of a given number of experiments. Statistical significance (evaluated using a one-way ANOVA with Dunett’s test) was acceptable to a level of *p* < 0.05. The concentration inducing a 50% decrease of cell viability as compared to control (IC_50_) was calculated using GraphPad Prism 7.03—nonlinear regression. 

## 3. Results

In the present study, the effect of four hop prenylflavonoids xanthohumol (XH), isoxanthohumol (IXH), 6-prenylnaringenin (6-PN), and 8-prenylnaringenin on the cell proliferation and efficacy of three cytostatics 5-fluorouracil (5-FU), oxaliplatin (OxPt), and irinotecan (IRT) in colorectal cancer cells in vitro was tested and evaluated. The effects of hop prenylflavonoids were compared to those of non-prenylated flavonoid naringenin (NAR).

### 3.1. Effect of Individual Prenylflavonoids and NAR on Cancer Cell Proliferation

At first, the effect of prenylflavonoids (XH, IXH, 6-PN, 8-PN) and NAR on the proliferation of human colorectal cancer cell lines (SW480, SW620, CaCo-2) was evaluated after 72 h of treatment. The CaCo-2 cells were used in a proliferating as well as differentiated state. When the CaCo-2 cells reach confluence, these cells are able to differentiate into enterocyte-like cells which express many transporters found in the enterocytes of the small intestine, forming tight junctions. The neutral red uptake assay was used to assess cell viability. 

**Figure 2:**
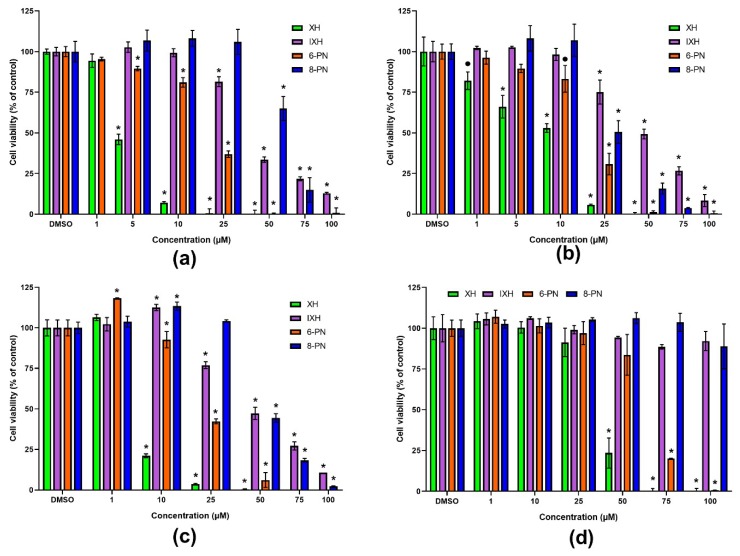
Effect of prenylflavonoids on the viability of SW480 (**a**), SW620 (**b**), proliferating CaCo-2 (**c**), and differentiated CaCo-2 cells (**d**) after 72 h of treatment. The cell viability was assayed using the neutral red uptake assay. Data are presented as a percentage of the respective control (= 100%) and represent the mean ± S.D. (*n* = 3) (• data with *p* < 0.01, * *p* < 0.001, one-way ANOVA with Dunett’s test).

All the prenylflavonoids as well as NAR caused the concentration-dependent inhibition of cancer cells proliferation (Figure 2), with XH being the most effective with an IC_50_ value 3.6 µM and 7.3 µM in SW480 and SW620 cells, respectively (Table 1). In contrast, NAR showed the lowest effect on the cell viability among tested compounds (IC_50_ > 200 µM in both cell lines). The sensitivity of cell lines to prenylflavonoids and NAR slightly differed, with XH and IXH showing higher antiproliferative potency in the SW480 cells, while 6-PN, 8-PN and NAR were more effective in the SW620 cells.

### 3.2. Effect of Individual Prenylflavonoids and NAR on the ROS Production in SW620 Cell Line

As is generally accepted, cancer cells generate more ROS than do normal cells and at the same time, they are sensitive to the effect of agents which augment oxidative stress and thus push these stressed cells beyond their limit [19]. Therefore, the pro-oxidative effect of prenylflavonoids and NAR was tested in the SW620 cells in order to elucidate the observed antiproliferative effects of these compounds. The SW620 cell line was chosen for further experiments, as most of the studied flavonoids showed higher antiproliferative activity in this cell line. ROS production was measured in the SW620 cells incubated with prenylflavonoids or NAR at a concentration equal to their IC_50_ using a DCFH-DA assay. ROS production was monitored during 24-hour incubation at several time points (0.5, 1, 4, 8, and 24 h).

**Figure 3:**
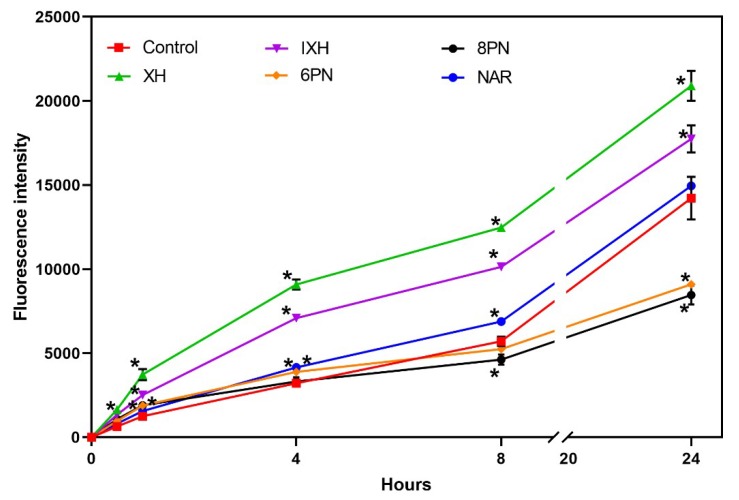
Effect of prenylflavonoids and naringenin on the ROS formation in SW620 cells. Data represent the mean ± S.D. (*n* = 3) (* data with *p* < 0.001, one-way ANOVA with Dunett’s test).

Compared to the control, all the studied prenylflavonoids caused a statistically significant (*p* < 0.001) increase in ROS production already within the first 30 min of incubation (Figure 3). XH and IXH exerted a strong pro-oxidative effect within the entire 24-hour incubation, while 6-PN and 8-PN initially elevated ROS levels in cells and then surprisingly displayed a strong antioxidant effect at the later intervals (8 and 24 hour of incubation). NAR showed only a mild pro-oxidative effect, which was statistically significant (*p* < 0.001) only at the intervals 4 h and 8 h. 

### 3.3. Effect of Prenylflavonoids and NAR on the Activity of Caspases in the SW620 Cell Line

In order to determine whether the observed antiproliferative activity of the prenylflavonoids and naringenin could be explained at least in part by the induction of apoptosis, the activities of initiator (caspase-8 and caspase-9) as well as effector caspases (caspases-3/7) were assessed in the SW620 cells, which were exposed to the studied compounds for 4, 8, and 24 h, with the activities of the caspases measured using a luminescence assay.

**Figure 4:**
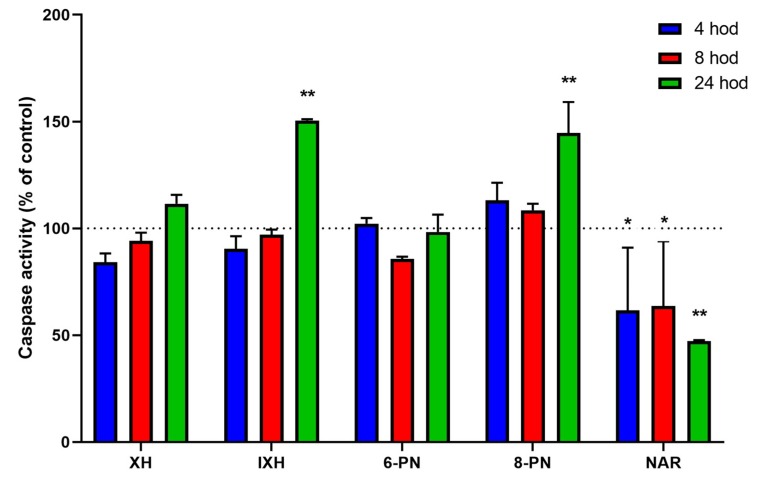
Effect of prenylflavonoids and naringenin on the activation of apoptosis in SW620 cells. The activity of caspases-3/7 was assessed after 4-, 8-, and 24-hour incubation. Data presented as percentage of untreated control (= 100%), represent the mean ± S.D. (*n* = 3) (* data with *p* < 0.05, ** *p* < 0.001, one-way ANOVA with Dunett’s test).

The activity of initiator caspases-8 and -9 were not significantly influenced by the studied compounds with the exception of NAR. This compound caused a decrease in caspase-9 activity by 19.7% compared to the untreated control (*p* < 0.001) after 4 h of exposure (data not shown). On the other hand, the activities of effector caspases-3/7 were markedly induced by IXH (50.5%) and 8-PN (44.7%) after 24-h exposure (*p* < 0.001). Surprisingly, NAR caused a significant inhibition (4 and 8 h: *p* < 0.05; 24 h: *p* < 0.001) of caspases-3/7 (by up to 52.7%) at all the chosen time intervals (Figure 4).

### 3.4. The Effect of Prenylflavonoids and NAR on Cytostatics’ Antiproliferative Activity in Colorectal Cancer Cell Lines

Subsequently, the effect of individual prenylflavonoids and naringenin on the antiproliferative activity of three commonly used CRC chemotherapeutics (i.e., 5-FU, IRT and OxPt) was studied in SW480 and SW620 cell lines. The IC_50_ values of these chemotherapeutics in SW480 and SW620 cells were as follows: 1.1 µM and 1.9 µM for OxPt, 1.7 µM and 25.4 µM for IRT, and 22.6 µM and 34.9 µM for 5-FU. According to combination index (CI) method recommendation [20], antiproliferative effects of drug combinations (cytostatic + prenylflavonoid) were tested at their constant ratios of IC_50_ values at which drugs are equipotent. The only exception represented NAR, which was due to its high IC_50_ and lower solubility tested at concentrations ranging from 75 to 200 µM. Data were analyzed using CalcuSyn software. Combination index CI < 1, CI = 1, and CI > 1 indicate a synergistic, additive and antagonistic effect, respectively. CI values calculated as a function of the fraction affected (Fa) = 0.8, which represents the percentage of growth inhibition (0.8 = 80%), are presented in Table 2. 

Synergistic effects were observed for 6-PN and IRT in both cell lines and for 6-PN and OxPt in SW620, while its combination with 5-FU and OxPt led to antagonistic activity in both cell lines and SW480, respectively. Synergistic to additive effects were observed for combinations of 8-PN with all three cytostatics in SW480, while antagonistic effects of these combinations were noticed in the SW620 cell line. Combination of NAR with all three cytostatics in both cell lines showed mostly a synergistic to additive effect except for the combination of NAR and 5-FU in SW480 cells, which exerted antagonistic activity. The only compound, which exerted antagonistic effects in combination with all three cytostatics in both cell lines, was IXH. This compound expressed strong antagonism with IRT (CI = 3.43) and OxPt (CI = 5.30) and moderate antagonism with 5-FU in SW620, while its effects ranged from moderate antagonism to antagonism in SW480. XH antagonized antiproliferative activity of 5-FU and OxPt in both cell lines, while its effect on the anticancer activity of IRT was additive and moderately antagonistic in SW480 and SW620, respectively.

### 3.5. Effect of 5-FU, OxPt and IRT and Their Combinations With Flavonoids on ROS Formation and Caspase Activity in SW620 Cell Line

Further, we decided to determine the influence of combinations of the studied flavonoids with cytostatics on ROS formation and the activity of caspases in SW620 cells. Combination effects of flavonoids and cytostatics were investigated at equipotent concentrations of the IC_50_ values for each compound. ROS production was monitored using a DCFH-DA assay at several time points (0.5, 1, 4, 8, and 24 h) during a 24-hour incubation. The activities of caspases-8, -9, and -3/7 were assessed using a luminescence assay kit at 4, 8, and 24 h of incubation.

None of the used cytostatics alone or in the combination with a prenylflavonoid caused an increase in ROS formation. In the case of NAR, its combinations with OxPt and IRI caused a feeble yet significant (*p* < 0.05) increase in ROS formation at all time points when compared to the control as well as to the NAR alone (data not shown).

Significant changes were found in the influence of individual compounds and their combinations on the activities of caspases-8, 9, and -3/7. All the cytostatics caused a small but significant increase (*p* < 0.05) in initiator caspase-8 and caspase-9 activity compared to the untreated control after 4-hour incubation. On the other hand, the activities of effector caspases-3/7 were not influenced by tested cytostatics at any time interval.

**Figure 5:**
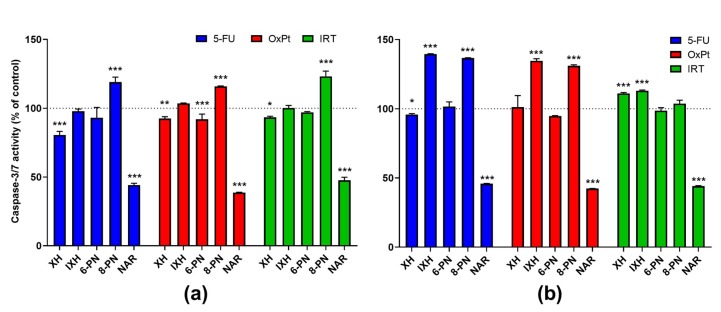
Effect of flavonoid–cytostatic combinations on the activation of apoptosis in SW620 cells. The activity of caspases 3/7 was assessed after 8-hour incubation (**a**) and 24-hour incubation (**b**). Data are presented as a percentage of respective cytostatic-treated control (= 100%) and represent the mean ± S.D. (*n* = 3) (* data with *p* < 0.05, ** *p* < 0.01, *** *p* < 0.001, one-way ANOVA with Dunett’s test).

Compared to the effect of cytostatics alone, the activity of caspase-8 was significantly increased in the combinations IXH-5-FU (*p* < 0.001), IXH-OxPT (*p* < 0.01), 8-PN-OxPt (*p* < 0.001), and 8-PN-IRT (*p* < 0.05) after 4-h incubation. In the case of caspase-9, the combinations IXH-5-FU (*p* < 0.01), 8-PN-OxPt (*p* < 0.001), and 8-PN-IRT (*p* < 0.01) were more potent than the respective cytostatics alone after 4-hour incubation. Compared to the effect of the prenylflavonoids alone, all the afore-mentioned combinations were more potent in the activation of initiator caspases. Regarding caspase-3/7 activity after 24-hour incubation, IXH with all three cytostatics (*p* < 0.001), 8-PN with 5-FU and OxPt (both *p* < 0.001), and XH with 5-FU (*p* < 0.05) and IRT (*p* < 0.001) caused a marked increase in their activity compared to the cytostatics alone, but these effects were significantly lower than (in case of IXH) or comparable to the effect of the respective flavonoids. Interestingly, NAR combined with all the cytostatics caused a marked induction (*p* < 0.05) in activities of the initiator caspases, while activities of the effector caspases were significantly suppressed (by up to 61.4%, *p* < 0.001) at all time intervals. The influence of flavonoid-cytostatic combinations on the activity of caspases-3/7 in SW620 cells after 8- and 24-hour incubation are presented in Figure 5.

## 4. Discussion

Beer, one of the most commonly consumed beverages, is rich in nutrient as well as non-nutrient components (e.g. minerals, vitamins, and phenolic compounds) and is the most important dietary source of prenylflavonoids. Some oncologists even recommend non-alcoholic beer as a source of liquids, energy and vitamin B in patients undergoing chemotherapy. Phenolic acids, prenylated chalcones and flavonoids, and proanthocyanidins and catechins (i.e., main beer polyphenolic compounds) originate from malt (70–80%) and from hops (20–30%). However, different beers vary in prenylflavonoids’ content considerably. The chemopreventive activity of beer and its constituents including prenylflavonoids have been studied and reported (reviewed in References [17,18]). Their ability to prevent the metabolic activation of procarcinogens to carcinogens due to the inhibition of cytochrome P450 1A enzymes [21] and to activate the detoxification enzyme, NAD(P)H:quinone oxidoreductase has been described [22,23], Moreover, the XH suppressed migration ability of cholangiocarcinoma cells [24], inhibited angiogenesis in pancreatic cancer cells in vitro as well as in vivo [15], and induced apoptosis in gastric cancer cells in vitro and in vivo [25]. 

The presented study was designed to evaluate the antiproliferative effect of four main hop-derived prenylflavonoids alone or in combination with three cytostatics commonly used to treat colorectal cancer in human colorectal cell lines (i.e., SW480, SW620, proliferating CaCo-2, and differentiated CaCo-2 cells). Based on the obtained results, the effect of these compounds on the ROS production and apoptosis induction was tested in the SW620 cells. All the studied activities of the prenylflavonoids were compared to those of non-prenylated flavonoid naringenin. 

All the studied prenylflavonoids inhibited cancer cell proliferation in a concentration-dependent manner, with XH being the most effective. The IC_50_ values for XH in SW480, SW620 and proliferating CaCo-2 cells are in low micromolar concentrations. The obtained IC_50_ values for XH and IXH in the SW620 cells were in good agreement with those previously reported by Hudcova et al. [26]. The IC_50_ values obtained for NAR were higher than 200 µM, which suggests that the presence of the prenyl group highly increases the antiproliferative effect of the flavonoids. Our results correspond well with previously published studies in which XH, 6-PN and 8-PN showed a marked antiproliferative effect in cancer cells, whereas NAR was found to be inactive [27,28]. As reported by Bartmanska et al. [27], the cytotoxic effects of prenylflavonoids are highly selective for cancer cells. Also in our experiments, the prenylflavonoids exerted much stronger cytotoxic effects towards proliferating CaCo-2 cells than towards differentiated CaCo-2 cells, which are widely accepted and used as a model of the intestinal epithelial barrier [29]. 

In order to elucidate the mechanism of the antiproliferative effects of the prenylflavonoids, two main hypotheses were considered: the modulation of intracellular oxidative stress and/or apoptosis activation. Cancer cells exhibit an increased basal level of ROS compared to normal cells; however, the level can be suppressed by an elevated activity of antioxidant enzymes in these cells. Therefore, therapeutic strategies that either augment ROS formation and/or suppress antioxidant defense mechanisms may push these stressed cells beyond their limit and thus reduce cancer progression [19]. The antioxidant properties of the main hop-derived prenylflavonoids have been described. These compounds act either directly as reducing antioxidants or indirectly via the induction of cellular antioxidant defense mechanisms [7,23]. However, the concentration- and time-dependent pro-oxidative effects of XH in several cancer cell lines have been documented [14,30,31]. For example, the XH induced transient superoxide anion radical formation in BHP-1 benign prostate hyperplasia cells, and thus triggered these cells into apoptosis [30]. In our experiments, XH and IXH showed a strong pro-oxidative effect in SW620 cells at all time intervals. Interestingly, 6-PN and 8-PN first caused a mild but significant increase in ROS levels, which was replaced by their marked antioxidant activity at later intervals of incubation. Blanquer-Rossello et al. [31] have reported the antioxidant activity of 8-PN in MCF-7 cells, but the authors measured ROS production only after 48-hour incubation and thus the initial 8-PN-mediated increase in ROS production as in our results could not be detected. In our experiments, the least expressed effect on ROS formation was shown in NAR, which exerted only a mild pro-oxidative effect. In a cell-free system, the strong pro-oxidative effect of this compound has been reported [7].

In addition to anti/pro-oxidative activity, some prenylflavonoids and NAR possess the ability to activate apoptosis in cancer cells [12,25,32]. Apoptosis caused by XH (20 µM) in T98G human malignant glioblastoma cell line was driven by ROS generation, which mediated the triggering of the mitochondrial pathway with Casp-9 and Casp-3 activation [14]. In our experiments, none of the prenylflavonoids influenced the activities of initiator Casp-8 and Casp-9, while IXH and 8-PN caused the significant activation of effector caspases-3/7. In 3T3-L1 adipocytes, XH and IXH caused an increase in Casp-3/7 activity, with XH being more effective [32], although concentrations of XH and IXH, which caused a significant change in Casp-3/7 activity, were 6.8-times and 1.7-times higher than those used in our experiments, respectively. In addition, 8-PN induced apoptosis in the MCF-7 cells trough activation of Casp-8 in a time- and concentration-dependent manner [33]. Surprisingly, NAR caused a significant reduction in Casp-9 activity after 4-h treatment and in Casp-3/7 activity at all time intervals. This discrepancy among our results and published data could be explained by the fact that NAR induces the apoptotic cascade only in the presence of estrogen receptor α/β [34], and SW620 cells do not express a detectable amount of estrogen receptor [35]. 

Based on the promising results of anti-proliferative and ROS-generating effects of prenylflavonoids, we sought to determine if these compounds are suitable candidates for anticancer combination therapy. The combination therapy, i.e., simultaneous administration of two or more drugs, has become a cornerstone in cancer therapy. This approach can potentially overcome drug resistance, reduce toxicity of the administered drugs, and increase their therapeutic effect [36]. Potentially, prenylflavonoids could represent suitable agents for combination therapy due to their antioxidant/pro-oxidant and pro-apoptotic activities. Moreover, the ability of IXH to enhance anticancer efficiency of paclitaxel in vivo as well as NAR ability to increase doxorubicin anti-tumor effect in vitro and in vivo have been reported [16,37]. Three chemotherapeutics (namely 5-fluorouracil, oxaliplatin and irinotecan), which represent the most commonly used drugs in conventional CRC treatment, were chosen for further experiments. 

While all prenylflavonoids alone were found to reduce the survival of SW480, SW620, and CaCo-2 cells, NAR was quite ineffective, which is in accordance with previous studies [26,27]. Surprisingly, the modulatory effect of prenylflavonoids on the cytotoxicity of 5-FU, IRT, and OxPt was mostly antagonistic, with the strongest antagonism found for combinations with OxPt. Moreover, IXH exerted antagonistic effects with all three chemotherapeutics, among which the anticancer efficiency of 5-FU was antagonized by XH, IXH, and 6-PN in both cell lines and by 8-PN in SW620 and NAR in the SW480 cells. The observed antagonistic effects of 6-PN and 8-PN with 5-FU could be partially explained by their antioxidant properties. As has been reported by Fu et al., the combination of 5-FU with antioxidants such as *N*-acetylcysteine blocks the ROS-dependent activation of Src, which results in decreased 5-FU-induced apoptosis [38]. Nevertheless, the synergism of 6-PN with OxPt and IRI does not corroborate this hypothesis; this synergism is likely caused by some other mechanism. Although the pro-oxidative and pro-apoptotic abilities of all three chemotherapeutics in cancer cells have been reported [39,40], only minor effects of these compounds on ROS formation and Casp-3/7 activation were observed in our experiments.

In conclusion, all the studied prenylflavonoids possess significant anticancer potential, a finding which was most apparent in the case of XH, which showed IC_50_ values ranging from 3.6 to 7.3 µM in proliferating colon cancer cells. These results are in agreement with the predominant view of beer as a chemopreventive beverage. As 6-PN significantly potentiated the antiproliferative effect of IRI in colorectal cancer cells, its potential use in combination therapy merits further study. On the other hand, XH and IXH, the most abundant prenylflavonoids in beer [17,18], antagonized the antiproliferative effects of 5-FU, OxPt, and IRI in colorectal cancer cells, thus these prenylflavonoids might impair the efficacy of chemotherapy and the consumption of beer (both alcoholic and non-alcoholic) should be avoided in patients undergoing 5-FU, OxPt and IRI treatment. In future, conclusions obtained in our in vitro experiments should be verified in vivo using mouse xenograft models supplemented with individual prenylflavonoids and/or beer in combination with chemotherapeutics.

## Figures and Tables

**Table 1:** IC_50_ values [µM] of NAR and prenylflavonoids (XH, IXH, 6-PN, 8-PN) in SW480, SW620 and CaCo-2 cells after 72 h of exposition.

Compound	SW480	SW620	CaCo-2 Proliferating	CaCo-2 Differentiated
XH	3.6	7.3	6.2	39.3
IXH	40.4	43.7	47.0	>100.0
6-PN	14.8	13.7	24.0	61.9
8-PN	56.3	24.9	48.9	>100.0
NAR	>200.0	>200.0	n.d.	n.d.

**Table 2:** Combination indexes (CI) of cytostatics (OxPt, 5-FU, IRT) and NAR, XH, IXH, 6-PN, and 8-PN in SW480 and SW620 after 72 h exposition. CIs were calculated as a function of the fraction affected (Fa), which represents the percentage of growth inhibition (0.8 = 80%) and was evaluated using the NRU assay.

Compound	SW480			SW620		
	5-FU	IRT	OxPt	5-FU	IRT	OxPt
XH	1.90	0.96	5.12	1.43	1.33	2.07
IXH	1.50	1.20	1.20	1.35	3.43	5.30
6-PN	4.80	0.86	3.20	1.30	0.88	0.38
8-PN	0.86	0.64	0.98	1.29	1.52	2.18
NAR	1.70	0.64	0.95	0.93	0.59	1.05

CI value <1, CI = 1, and CI > 1 indicates synergism, additive effect, and antagonism, respectively.

## References

[1] Ferlay J., Soerjomataram I., Dikshit R., Eser S., Mathers C., Rebelo M., Parkin D.M., Forman D., Bray F. (2015). Cancer incidence and mortality worldwide: Sources, methods and major patterns in GLOBOCAN 2012. Int. J. Cancer.

[2] Sag A.A., Selcukbiricik F., Mandel N.M. (2016). Evidence-based medical oncology and interventional radiology paradigms for liver-dominant colorectal cancer metastases. World J. Gastroenterol..

[3] Wilson T.R., Johnston P.G., Longley D.B. (2009). Anti-apoptotic mechanisms of drug resistance in cancer. Curr. Cancer Drug Targets.

[4] Hammond W.A., Swaika A., Mody K. (2016). Pharmacologic resistance in colorectal cancer: A review. Ther. Adv. Med. Oncol..

[5] Prochazkova D., Bousova I., Wilhelmova N. (2011). Antioxidant and prooxidant properties of flavonoids. Fitoterapia.

[6] Rossi M., Bosetti C., Negri E., Lagiou P., La Vecchia C. (2010). Flavonoids, proanthocyanidins, and cancer risk: A network of case-control studies from Italy. Nutr. Cancer.

[7] Miranda C.L., Stevens J.F., Ivanov V., McCall M., Frei B., Deinzer M.L., Buhler D.R. (2000). Antioxidant and prooxidant actions of prenylated and nonprenylated chalcones and flavanones in vitro. J. Agric. Food Chem..

[8] Gerhauser C. (2005). Broad spectrum anti-infective potential of xanthohumol from hop (Humulus lupulus L.) in comparison with activities of other hop constituents and xanthohumol metabolites. Mol. Nutr. Food Res..

[9] Legette L.L., Luna A.Y., Reed R.L., Miranda C.L., Bobe G., Proteau R.R., Stevens J.F. (2013). Xanthohumol lowers body weight and fasting plasma glucose in obese male Zucker fa/fa rats. Phytochemistry.

[10] Dorn C., Massinger S., Wuzik A., Heilmann J., Hellerbrand C. (2013). Xanthohumol suppresses inflammatory response to warm ischemia-reperfusion induced liver injury. Exp. Mol. Pathol..

[11] Erkkola R., Vervarcke S., Vansteelandt S., Rompotti P., De Keukeleire D., Heyerick A. (2010). A randomized, double-blind, placebo-controlled, cross-over pilot study on the use of a standardized hop extract to alleviate menopausal discomforts. Phytomedicine.

[12] Pan L., Becker H., Gerhauser C. (2005). Xanthohumol induces apoptosis in cultured 40-16 human colon cancer cells by activation of the death receptor- and mitochondrial pathway. Mol. Nutr. Food Res..

[13] Sun Z., Zhou C., Liu F., Zhang W., Chen J., Pan Y., Ma L., Liu Q., Du Y., Yang J. (2018). Inhibition of breast cancer cell survival by Xanthohumol via modulation of the Notch signaling pathway in vivo and in vitro. Oncol. Lett..

[14] Festa M., Capasso A., D’Acunto C.W., Masullo M., Rossi A.G., Pizza C., Piacente S. (2011). Xanthohumol induces apoptosis in human malignant glioblastoma cells by increasing reactive oxygen species and activating MAPK pathways. J. Nat. Prod..

[15] Saito K., Matsuo Y., Imafuji H., Okubo T., Maeda Y., Sato T., Shamoto T., Tsuboi K., Morimoto M., Takahashi H. (2018). Xanthohumol inhibits angiogenesis by suppressing nuclear factor-kappaB activation in pancreatic cancer. Cancer Sci..

[16] Krajnovic T., Kaluderovic G.N., Wessjohann L.A., Mijatovic S., Maksimovic-Ivanic D. (2016). Versatile antitumor potential of isoxanthohumol: Enhancement of paclitaxel activity in vivo. Pharmacol. Res..

[17] Gerhauser C. (2005). Beer constituents as potential cancer chemopreventive agents. Eur. J. Cancer.

[18] Stevens J.F., Page J.E. (2004). Xanthohumol and related prenylflavonoids from hops and beer: To your good health!. Phytochemistry.

[19] Galadari S., Rahman A., Pallichankandy S., Thayyullathil F. (2017). Reactive oxygen species and cancer paradox: To promote or to suppress?. Free Radic. Biol. Med..

[20] Chou T.C. (2006). Theoretical basis, experimental design, and computerized simulation of synergism and antagonism in drug combination studies. Pharmacol. Rev..

[21] Plazar J., Zegura B., Lah T.T., Filipic M. (2007). Protective effects of xanthohumol against the genotoxicity of benzo(a)pyrene (BaP), 2-amino-3-methylimidazo[4,5-f]quinoline (IQ) and tert-butyl hydroperoxide (t-BOOH) in HepG2 human hepatoma cells. Mutat. Res..

[22] Yu L., Zhang F., Hu Z., Ding H., Tang H., Ma Z., Zhao X. (2014). Novel prenylated bichalcone and chalcone from Humulus lupulus and their quinone reductase induction activities. Fitoterapia.

[23] Dietz B.M., Kang Y.H., Liu G., Eggler A.L., Yao P., Chadwick L.R., Pauli G.F., Farnsworth N.R., Mesecar A.D., van Breemen R.B. (2005). Xanthohumol isolated from Humulus lupulus Inhibits menadione-induced DNA damage through induction of quinone reductase. Chem. Res. Toxicol..

[24] Jongthawin J., Techasen A., Loilome W., Yongvanit P., Namwat N. (2012). Anti-inflammatory agents suppress the prostaglandin E2 production and migration ability of cholangiocarcinoma cell lines. Asian Pac. J. Cancer Prev..

[25] Guo D., Zhang B., Liu S., Jin M. (2018). Xanthohumol induces apoptosis via caspase activation, regulation of Bcl-2, and inhibition of PI3K/Akt/mTOR-kinase in human gastric cancer cells. Biomed. Pharmacother..

[26] Hudcova T., Bryndova J., Fialova K., Fiala J., Karabin M., Jelinek L., Dostalek P. (2014). Antiproliferative effects of prenylflavonoids from hops on human colon cancer cell lines. J. Inst. Brew..

[27] Bartmanska A., Tronina T., Poplonski J., Milczarek M., Filip-Psurska B., Wietrzyk J. (2018). Highly Cancer Selective Antiproliferative Activity of Natural Prenylated Flavonoids. Molecules.

[28] Stompor M., Uram L., Podgorski R. (2017). In Vitro Effect of 8-Prenylnaringenin and Naringenin on Fibroblasts and Glioblastoma Cells-Cellular Accumulation and Cytotoxicity. Molecules.

[29] Angelis I.D., Turco L. (2011). Caco-2 cells as a model for intestinal absorption. Curr. Protoc. Toxicol..

[30] Strathmann J., Klimo K., Sauer S.W., Okun J.G., Prehn J.H., Gerhauser C. (2010). Xanthohumol-induced transient superoxide anion radical formation triggers cancer cells into apoptosis via a mitochondria-mediated mechanism. FASEB J..

[31] Blanquer-Rossello M.M., Oliver J., Valle A., Roca P. (2013). Effect of xanthohumol and 8-prenylnaringenin on MCF-7 breast cancer cells oxidative stress and mitochondrial complexes expression. J. Cell. Biochem..

[32] Yang J.Y., Della-Fera M.A., Rayalam S., Baile C.A. (2007). Effect of xanthohumol and isoxanthohumol on 3T3-L1 cell apoptosis and adipogenesis. Apoptosis.

[33] Brunelli E., Minassi A., Appendino G., Moro L. (2007). 8-Prenylnaringenin, inhibits estrogen receptor-alpha mediated cell growth and induces apoptosis in MCF-7 breast cancer cells. J. Steroid Biochem. Mol. Biol..

[34] Totta P., Acconcia F., Leone S., Cardillo I., Marino M. (2004). Mechanisms of naringenin-induced apoptotic cascade in cancer cells: Involvement of estrogen receptor alpha and beta signalling. IUBMB Life.

[35] Nguyen-Vu T., Wang J., Mesmar F., Mukhopadhyay S., Saxena A., McCollum C.W., Gustafsson J.A., Bondesson M., Williams C. (2016). Estrogen receptor beta reduces colon cancer metastasis through a novel miR-205—PROX1 mechanism. Oncotarget.

[36] Bayat Mokhtari R., Homayouni T.S., Baluch N., Morgatskaya E., Kumar S., Das B., Yeger H. (2017). Combination therapy in combating cancer. Oncotarget.

[37] Zhang F.Y., Du G.J., Zhang L., Zhang C.L., Lu W.L., Liang W. (2009). Naringenin enhances the anti-tumor effect of doxorubicin through selectively inhibiting the activity of multidrug resistance-associated proteins but not P-glycoprotein. Pharm. Res..

[38] Fu Y., Yang G., Zhu F., Peng C., Li W., Li H., Kim H.G., Bode A.M., Dong Z., Dong Z. (2014). Antioxidants decrease the apoptotic effect of 5-Fu in colon cancer by regulating Src-dependent caspase-7 phosphorylation. Cell Death Dis..

[39] Huang Y.F., Zhu D.J., Chen X.W., Chen Q.K., Luo Z.T., Liu C.C., Wang G.X., Zhang W.J., Liao N.Z. (2017). Curcumin enhances the effects of irinotecan on colorectal cancer cells through the generation of reactive oxygen species and activation of the endoplasmic reticulum stress pathway. Oncotarget.

[40] Mhaidat N.M., Bouklihacene M., Thorne R.F. (2014). 5-Fluorouracil-induced apoptosis in colorectal cancer cells is caspase-9-dependent and mediated by activation of protein kinase C-delta. Oncol. Lett..

